# Research on the coordinated measurement and spatiotemporal evolution of digital economy empowering ecotourism

**DOI:** 10.1371/journal.pone.0323723

**Published:** 2025-05-27

**Authors:** Yuan Wang, Yuxin Li, Linling Zheng, Yihua Zhang

**Affiliations:** 1 School of Business Administration, Jimei University, Xia Men, Fujian, China; 2 School of Fuzhou Polytechnic, Fuzhou, Fujian, China; University of Naples Federico II Department of Business Economics: Universita degli Studi di Napoli Federico II Dipartimento di Economia Management e Istituzioni, ITALY

## Abstract

Although the internet economy in China has had enormous growth in recent years, the country’s ecotourism business has shown a far slower rate of expansion. There is an immediate need to discuss how to leverage the digital economy to advance ecotourism and achieve synchronized development between the two. The coupling and coordination between China’s digital economy and ecotourism are examined in this study using a coupling coordination model to evaluate their temporal and geographical growth. Panel data that was gathered from 30 provinces between 2011 and 2022 served as the basis for the research. The historical and geographical evolution of their attributes is examined by the application of standard deviational ellipse analysis, kernel density estimation, and Dagum Gini coefficient. The results show a steady improvement in the degree of connectivity and synchronization between the digital economy and ecotourism. This improvement follows a general geographical trend of being robust in the eastern regions and feeble in the western regions, while being prominent in the southern areas and less significant in the northern areas. The primary cause of the unequal growth of the digital economy and ecotourism in various places is regional inequities, as evidenced by the variable drop in the Gini coefficient of coupling coordination degree. A distinct polarization tendency in the national, eastern, and western areas, along with an annual expansion of the right tail in the national kernel density, cause the kernel density curve to continuously move to the right. The standard deviational ellipse shows that the geographical inequalities between ecotourism and the digital economy are gradually diminishing. The three primary regions are seeing a dynamic reduction in the size of the ellipse, and the distribution of places is becoming more focused. The article suggests enhancing digital transformation in ecotourism and promoting industry growth for better-coordinated development.

## Introduction

In the 21st century, China has become the world’s second-largest digital economy, signifying the onset of the digital era. Based on data from the China Academy of Information and Communications Technology, it is projected that by 2023, China’s digital economy will expand to 56.1 trillion yuan, seeing an annual growth rate of around 11.75%. The digital economy contributes a significant proportion to the GDP, comparable to that of the secondary industry. It accounts for almost 40% of the national economy. The process of digital industrialization is currently transitioning from a state of incremental growth to a state of significant improvement in quality. In China’s new era of economic development, the progress of the digital economy has emerged as a critical strategy and an inevitable trend, as evidenced by the policies and measures that other nations are putting in place to support its growth. This creates a favorable policy environment and technological support for the digital progress of the tourism industry. The “14th Five-Year” Tourism Development Plan highlights the importance of advancing the new wave of industrial and technological revolutions, implementing a strategy focused on innovation-driven development, pursuing sustainable development, enhancing the coordination mechanism in the field of ecological civilization, promoting a comprehensive shift towards a green economy and society, and expediting the growth of intelligent tourism characterized by digitalization, networking, and intelligence. Ecotourism, on the other hand, was defined in 1990 by the International Ecotourism Society (IES) as a type of tourism in natural areas that protects the environment and improves the well-being of the inhabitants [1], and promotes national and regional development more than the traditional model of tourism development [2]. Ecotourism is seeing new chances for growth, as the tourism industry becomes a prominent example of the platform economy within the digital economy. Therefore, the attainment of a harmonized fusion between the digital economy and ecotourism has become an unavoidable trajectory and will significantly contribute to the construction of a picturesque China.

Currently, as the world’s second-largest digital economy, China’s ecotourism serves as a “green engine” for industrial structure upgrading, a synergistic node between digital economy and ecological economy, and a crucial domain for consumption upgrading and domestic demand expansion. However, the digital transformation of ecological tourism faces multiple challenges. Fragmented resources and insufficient industrial chain coordination hinder comprehensive data interoperability in digital tourism platforms, resulting in “data silos.” The regional disparity in digital technology application exacerbates developmental imbalances, creating a “digital divide.” Underlying these issues are more complex systemic barriers: factor market segmentation and path dependence caused by the inertia of traditional tourism development models, the tension between technological innovation and institutional constraints due to regulatory lag, and the transmission effects of interregional factor flow barriers and urban-rural dual structures that intensify regional economic imbalances.

These challenges not only impede the realization of smart tourism objectives outlined in the “14th Five-Year Plan for Tourism Development” but also potentially obstruct the implementation of the dual-carbon (peak carbon and carbon neutrality) strategy in the tourism sector, holding critical significance for China’s ongoing economic transformation and sustainable development agenda. Current international research exhibits notable limitations in addressing these complex issues. While some scholars emphasize the importance of intelligent technologies for sustainable ecotourism, there remains a paucity of cross-scale analyses integrating digital economy and ecological tourism [3]. Similarly, Sgroi’s research on digital technologies’ role in promoting sustainable mountain tourism, while valuable, focuses on specific geographical contexts and fails to address structural barriers in diverse tourism destinations’ digital transformation [4].

This study contributes value by evaluating the coupling coordination between China’s digital economy and ecotourism, proposing scientifically grounded strategies for digital transformation. It facilitates a virtuous interplay between technological advancement and ecological preservation to achieve high-quality tourism development. China’s successful digital transformation in ecotourism not only advances the greening and decarbonization of its economy, society, and tourism industry but also fosters widespread adoption of sustainable tourism practices. These achievements offer valuable lessons for global counterparts. The research reveals universal implications from China’s case, providing replicable digital transformation pathways for global ecotourism. The proposed digital analytics approaches enhance tourism resource utilization efficiency, offering immediate applicability for regions facing overtourism pressures (e.g., Mediterranean coastal areas). Moreover, the regional balanced development measures present innovative solutions to the “digital divide” challenge prevalent in developing countries.

## Related works

### Digital economy

Canadian researcher Don Tapscott established the concept of the “digital economy” in 1996 [5]. Based on the fundamentals of science and technology, the digital economy has grown significantly. In the domain of the digital economy, there are regular and concentrated advancements in science and technology. The connotation and extension of the digital economy has evolved over time, and the scope of application of digital technology has gradually expanded. The utilization of digital technology significantly decreases the expenses associated with searching, entering, transporting, and reproducing information, hence uncovering limitless possibilities for improving economic efficiency [6]. In general, scholars’research on the digital economy has focused on the following two areas:

First, the digital economy helps to promote green and low-carbon development and long-term sustainable development. Studies have shown that the development of the digital economy promotes the transformation and upgrading of urban industrial infrastructure. Tao et al. believe that the digital economy accelerates information transfer and enriches entrepreneurial resources, promotes high-quality development by enhancing entrepreneurial vitality, and encourages public entrepreneurship, which is an important mechanism for the digital economy to release the dividends of high-quality development, and the positive impacts of the digital economy have the spatial spillover characteristics of non-linear incrementalism and the “marginal effect”, which promotes the sustainable development of the city [7]. Nochta, T. argues that the application of digital technology to urban management can not only improve the operational efficiency of cities through data management, but also stimulate the transformation of urban residents’ lifestyles to low-carbon and green, and reduce carbon emissions in the digitalization of cities [8].

Second, the digital economy effectively promotes scientific and technological innovation. Hence, digital technology imparts unique attributes to the digital era that are clearly distinct from those of the industrial age: First and foremost, digital technology has eliminated the restrictions imposed by existing hierarchies and technological constraints, leading to the fast emergence of diverse business models. Furthermore, it reduces the obstacles for firms to penetrate the market [9], enabling them to build links with external marketplaces in order to reduce expenses [10], thereby leading to a rise in the prevalence of “disruptive” business innovations. Xie et al. found a significant positive correlation between the application of digital technology and manufacturing change, especially in low-tech intensive industries that are more sensitive to digital technology [11]. Hao et al. argued that digitization has had a far-reaching impact on China’s economy and society, driving digital technological innovations, improving productivity, and guiding society towards a more inclusive and knowledge-driven environment [12].

### Ecotourism

Ecotourism began to appear in academic research in the late 1980s, and was recognized by Ceballos-Lascuráin as a special form of nature-based tourism that maintains local ecological levels while preserving the environment and providing visitors with a comfortable experience of nature and humanity [13,14]. In 1990, the International Ecotourism Society (IES) defined ecotourism as a form of tourism that takes place in a natural region to protect the environment and improve the welfare of the residents [1]. Ecotourism as an emerging topic has gradually become mature after years of research and development, and countless scholars have defined and dissected it. The purpose of ecotourism is to promote long-term sustainable development, which includes, but is not limited to, the conservation of natural resources [15], the enhancement of local economic incomes [16] and the promotion of gender equality [17]. The advancement of the conventional tourism sector frequently resulted in ecological harm and inefficient use of resources [18], necessitating the shift towards a high-quality ecotourism model as an unavoidable option for sustainable development. Advocating for high-quality ecotourism involves not only promoting a balanced relationship between humans and nature but also has a substantial impact on increasing socio-cultural consciousness [19] and safeguarding resources and the environment [20]. Ecotourism development also contributes to the growth and reinforcement of sectors that prioritize resource conservation and environmental sustainability. It serves as a crucial pathway for industrial transformation and urbanization.

However, with the low-carbon and green development of society, ecotourism is gradually becoming more common and the number of tourists is increasing year by year [21]. Some scholars have then found that ecotourism highlights some urgent problems: the lack of appropriate infrastructure construction to meet the excess of tourists, the lack of appropriate protection and respect for the local ecological and cultural environment and so on [22]. In this context, Niñerola et al. conducted a study of papers on sustainable tourism development in Scopus from 1987–2018 using bibliometric methods and VOSviewer [23]. Fu used SWOT analysis to analyze the strengths and weaknesses, opportunities and threats of ecotourism development in a certain region, and proposed that the region should combine the development status of tourism, economy and environment to determine scientific and reasonable ecotourism development principles [24]. Li believes that the development of ecotourism needs to be integrated with green technological innovation, which is an important guarantee for ecotourism to really return to the ecological trajectory and industrial transformation and upgrading, and is conducive to the sustainable development of ecotourism [25].

### Study on the correlation between digital economy and ecotourism

Existing research on the subject of the linkage between the digital economy and ecotourism development is broadly divided into two lines of thought: first, the direct impact of the characteristics of the digital economy itself on ecotourism; and second, the indirect impact of the digital economy through changes in inputs of factors of production, upgrading of the industrial structure, and improving the efficiency of resource allocation. However, there is less relevant literature on the degree of synergistic development between the two.

The direct impact of digital technology on the ecosystem is mainly mentioned in: De Zoysa, M. Through the study, it was found that ecotourism enhances the conservation activism of the tourists through interactive and information-rich activity experiences, which continuously increases the green and low-carbon awareness. And as the educated population continues to grow, ecological awareness gradually increases [26]. In addition, ecotourism has a strong reliance on data-driven decision-making, planning, and performance evaluation, where tourism practitioners collect and analyze data to draw conclusions about relevant improve the sustainability and profitability of the tourism industry, and adopting these innovations to improve the status quo contributes to environmental stewardship and cultural preservation [27]. Ecotourism has always sought a dynamic balance between preserving the ecological integrity of a destination and meeting the personalized experiences of tourists. Ecotourism aims to maintain local ecological balance and continually improve natural resilience by reducing carbon emissions, protecting habitats and curbing overexploitation of natural resources [28]. KC et al. argued that responsible tourism practices similar to the establishment of protected areas and encouraging responsible behavior among tourists help to conserve biodiversity and ecosystems. By doing so, destinations reduce their environmental impacts and protect natural resources and rare and endangered animals, so it is crucial to follow through on environmentally conscious actions in all parts of the tourism value chain [29]. Li et al. conducted a fixed-effects regression analysis on panel data from 160 countries from 2005–2016, and found that there is an inverted-u nonlinear and digital economy have an inverted u-shaped nonlinear relationship, i.e., the digital economy has a positive effect on carbon emissions [30].

The indirect impacts of digital technologies on ecosystems are mainly mentioned in: Alsahafi et al. argued that ecotourism websites are designed to increase tourists’ well-being, loyalty, and ecologically conscious behaviors by catering to the personalized needs of different customers, and that ecotourism can be made sustainable by using digital technologies including measures to rationalize the allocation of tourism resources, enrich the tourists’ experience, and protect the environment [31]. Dharmayanti et al. showed that ecotourism ecosystems are influenced by IoT, artificial intelligence and data analytics, and that these digital technologies promote sustainable practices, sustained engagement, and personalized participation, and that the tourism sector can leverage these digital technologies to achieve more sustainable, inclusive, and environmentally friendly changes [32]. Dong et al. argued that the digital economy has transformed the traditional development model into a sustainable development model, saving limited resources, improving energy efficiency, optimizing the economic structure, adjusting the industrial structure and energy structure, constructing an economic pattern of green, low-carbon and circular development, and ultimately achieving coordinated development of climate action and contributing to the protection of the environment [33]. The deep integration and innovative application of digital technology in the fields of energy, resources and environment have made the traditional industries increasingly motivated to improve the innovation capacity of digital technology, giving rise to the emergence of green development models. The digital economy helps to promote the green and low-carbon development of tourism and the sustainable development of ecotourism. Kong and Li’s research at the city level found that the development of the digital economy helps optimize the eco-efficiency of cities [34].

In terms of the synergistic relationship between digital technology and the ecological environment, although there is a considerable amount of research in the academic community on ecological conservation, the digital economy, and cultural-tourism integration, these studies have mainly focused on the interconnections between the two [35]. China’s tourism business has recently undergone a new era of digital advancement. Navío-Marco and other researchers have highlighted that the tourism business is the most prolific sector in terms of online sales of items and services [36]. Furthermore, the advent of digital technology, particularly sharing platforms, has revolutionized the entire value chain of the conventional tourism industry. The emergence of the digital economy introduces novel input factors, enhances resource allocation efficiency, and boosts total factor productivity, hence creating new opportunities for the advancement of tourism products [37]. Information and communication technology has provided strong support to the tourist sector, allowing online platforms to expand quickly and introducing new tools for marketing and administration [38]. Several scholars have verified, using state space models and vector autoregression models, that the degree of informatization has a progressively favorable influence on long-term tourism development. However, in the short term, tourism development does not stimulate economic growth. At a small scale, the process of incorporating information technology into rural tourism is closely connected to the expansion of the tourism sector. Actually, to some extent, the informatization of rural tourism contributes to the advancement of the regional tourism sector [39].

In conclusion, the above literature emphasizes the multifaceted nature of the relationship between the digital economy and tourism, providing important theoretical support and research indicators for the coordinated development of informatization and tourism in China. The studies described above offer substantial theoretical backing and research indications for the synchronized advancement of informatization and tourism in China. However, the majority of the work that is now available examines the relationship between informatization and the coordinated expansion of the tourist sector, with little attention paid to the coordinated expansion of the digital economy and ecotourism. Investigations frequently focus on one viewpoint, analyzing how the digital economy impacts tourism development, while overlooking the influence of the expansion of tourism on the digital economy. Thus, more work has to be done to better understand how the digital economy and the growth of ecotourism are intertwined. The interdependence between the digital economy and ecotourism forms a coupled relationship that exhibits consistent development and change to a certain extent, but it remains a challenge to accurately quantify the degree of coordination between the two in order to judge the trend of spatial and temporal evolution.

## Materials and methods

### Data collection

The National Bureau of Statistics, the “China Tourism Statistics Yearbook,” the “China Culture and Tourism Statistics Yearbook,” the People’s Republic of China’s Ministry of Culture and Tourism, and a number of provincial statistics yearbooks were the primary sources of data. During the data compilation process, a tiny amount of missing data was filled in using the mean replacement approach.

### Construction of the index system

It is imperative to build an objective and fair indicator assessment system that can monitor the advancement of both the digital economy and ecotourism scientifically in order to assess the growth rates of both industries. This article presents an assessment framework of growth indicators for ecotourism and the digital economy, grounded in a thorough understanding of their fundamental meanings and drawing on the academic accomplishments of earlier studies. This study employs a composite assessment model to quantitatively quantify the integrated development levels of ecotourism and the digital economy. It does this by using the entropy weight technique to determine the weights of numerous factors.

An ecotourism and digital economy indicator system is presented in this research. Six core indicators and sixteen supplementary indicators make up the two tiers of the system. The digital economy, ecotourism, and economic development are all covered by these metrics in different ways. Experts in the relevant disciplines were consulted using the Delphi approach to minimize the influence of personal biases and enhance the impartiality and dependability of the evaluation findings. Furthermore, the weights of the indicators in the assessment framework for the digital economy and ecotourism were established using the entropy weight technique. The entropy weight method avoids the subjective bias problem of the Analytic Hierarchy Process (AHP) relying on expert experience in emerging interdisciplinary fields such as digital economy and ecotourism [40]. Moreover, ecotourism data often exhibits spatial heterogeneity (such as “strong in the east and weak in the west” characteristics), and the assumptions of normal distribution and linear relationship in principal component analysis (PCA) may reduce its explanatory power [41].

In the end, sixteen indicators from thirty provinces—excluding Tibet, Hong Kong, and Macao—were subjected to standardized processing for the years 2011–2022. These thirty provinces were further divided into eastern, central, and western regions [42–44]. The weight values of each indication were ascertained using the entropy weight approach; Table 1 provides specifics.

**Table 1:** Indicator evaluation system and indicator weights for digital economy and ecotourism.

System layers	First-level indicators	Secondary indicators	Variable	Unit	Indicator attributes	First-level indicator weights	Secondary indicator weights
A. Digital Economy	Digital infrastructure	Number of postal offices	X_1_	items	+	0.3904	0.1449
		Internet broadband access ports per capita	X_2_	items/ person	+		0.0812
		Length of fiber optic cable lines per capita	X_3_	km^2^/ 10,000 person	+		0.1023
		Telephone penetration rate per capita	X_4_	items/ 100 person	+		0.0620
	Digital industrialization	R&D expenditure per capita	X_5_	CNY/ person	+	0.3082	0.1803
		R&D expenditure intensity	X_6_	%	+		0.1279
	Degree of digital development	Number of patent applications granted per capita	X_7_	pieces/ 10,000 person	+	0.3014	0.2441
		Digital Financial Inclusion Index	X_8_	–	+		0.0573
B. Ecotourism	Tourism benefit	Total tourism revenue	X_9_	100 million CNY	+	0.3727	0.1673
		Domestic tourist number	X_10_	100 million person	+		0.2054
	Tourism resource	Number of travel agencies	X_11_	household	+	0.4248	0.1381
		Number of tourist attractions	X_12_	items	+		0.1539
		Number of museums	X_13_	items	+		0.1328
	Ecological quality	Industrial SO_2_ emissions	X_14_	10,000 t	–	0.2025	0.0239
		Park green space per capita	X_15_	m^2^/ person	+		0.0592
		Forest coverage rate	X_16_	%	+		0.1194

### Methods

#### Coupling coordination degree models.

(1) Entropy Weight Method

To ensure that the obtained data is not influenced by human factors and to maintain its objectivity and scientific validity, this study employs the entropy method to determine the specific weight ratios of each indicator [45,46]. The procedure is as follows:

Standardization process:

For positive indicators, the standardization formula is as follows:


$$Y_{ij}=0.1+0.9\frac{x_{ij}-min\left(x_{j}\right)}{\max\left(x_{j}\right)-min\left(x_{ij}\right)}$$ (1)

For negative indicators, the standardization formula is as follows:


$$Y_{ij}=0.1+0.9\frac{\max\left(x_{j}\right)-x_{ij}}{\max\left(x_{j}\right)-min\left(x_{j}\right)}$$ (2)

The formula for calculating the indicator weights is as follows:


$$E_{i}=-\frac{1}{\ln(n)}\sum_{i=1}^{m}p_{ij}\ln p_{ij}$$ (3)

The calculation formula for P value is as follows:


$$P_{ij}=-\frac{Y_{ij}}{\sum_{i=1}^{n}Y_{ij}}$$ (4)

The entropy method is used to calculate the information entropy values of the standardized data. $E_{ij}$ refers to the proportion under the jth indicator of province i, $Y_{ij}$ is the standardized value of the jth indicator raw data for province i, and n is the number of evaluation objects.

The redundancy of each indicator is calculated as follows:


$$G_{j}=1-E_{j}$$ (5)

The weights of each evaluation indicator are calculated as follows:


$$W_{j}=\frac{G_{j}}{\sum_{j=1}^{n}G_{j}}$$ (6)

The comprehensive development level of the digital economy and ecological tourism is calculated using the following formula:


$$U_{i}=\sum_{j=1}^{m}{W_{j}Y}_{ij}$$ (7)

(2) Coupling degree model construction

The purpose of this research is to create a coupling degree model for the ecotourism and digital economy systems. It investigates the level of interdependence and coordinated development between these two subsystems, highlighting their dynamic connections and mutually beneficial interactions. Utilizing the academic work of some scholars [47–49], the procedures for calculating the degree of coupling are outlined according to Equations (8) and (9).


$$C=\sqrt{\frac{u_{1}.u_{2}}{{[\frac{u_{1}+u_{2}}{2}]}^{2}}}$$ (8)

$$u_{i}=\sum_{j=1}^{m}w_{ij}x_{ij}$$ (9)

The degree of interdependence between ecotourism systems and the digital economy is indicated by variable C. The coupling degree C is a numerical value that falls within the range of 0–1. A larger value of C signifies a stronger amount of connection between the two subsystems, indicating a higher degree of interdependence. The evaluation metrics of the ecotourism and digital economy systems are reflected in the variables u_1_ and u_2_, respectively. The variable W_ij_ is the weight of the jth indicator for i. The variable $x_{ij}\;$is the standardized data value of the jth indicator for i. M represents the number of evaluation indicators, ranging from 0 to 30.

(3) Coupling Coordination Degree Model Construction

A comprehensive evaluation is carried out to appraise the degree of synchronization and advancement in growth between the ecotourism and digital economy subsystems. The equations for determining the coupling coordination degree, based on the study findings by some scholars [50–52], are provided in equations (10) and (11). In these equations, C represents the coupling degree and T represents the coordination index.


$$T_{ij}={\alpha U}_{i}+{\beta U}_{j}$$ (10)

$$D_{ij}={\left(C_{ij}\cdot T_{ij}\right)}^{\frac{1}{2}}$$ (11)

The variable $T_{ij}$ represents the comprehensive evaluation value between the digital economy and the ecotourism system. It is obtained by weighted summation of the individual indicators of both systems. α is the weight coefficient representing the importance of the digital economy system $U_{i}$, and $U_{i}$ is the evaluation index value of the digital economy system. β is the weight coefficient representing the importance of the digital economy system $U_{j}$, and $U_{j}$ is the evaluation index value of the ecotourism system.

$C_{ij}\;$represents the coupling degree between the digital economy and ecotourism, which measures the interdependence between the two systems. A larger value indicates a stronger relationship. A greater value of D signifies enhanced cooperation between the two systems. D represents the effect weights of the digital economy and ecotourism, respectively. This article assumes that the digital economy and ecotourism are given equal importance, with a weight of 0.5 each. Based on earlier studies on the coupling degree measuring methodology, Table 2 usually displays the coupling coordination degree categorization criteria.

**Table 2:** Ranking the coupling coordination of digital economy and ecotourism.

Coupling Coordination Degree	Coupling Effect Level
(0,0.4]	Low coupling coordination
(0.4,0.6]	Medium coupling coordination
(0.6,0.8]	High coupling coordination
(0.8,1)	Extreme coupling coordination

#### Methods of analysing spatial and temporal evolution.

This work investigates the spatiotemporal evolution properties of coupling coordination using the Dagum Gini coefficient. It examines the origins of the intra- and interregional relative differences as well as the overall relative disparities in coupling coordination. Furthermore, the temporal evolution features of coupling coordination are analyzed using kernel density estimation, and the spatial evolution characteristics of coupling coordination are investigated using standard deviational ellipse analysis.

(1) Dagum Gini coefficient method

In order to evaluate the overall relative discrepancies in the coupling coordination between China’s digital economy and ecotourism, this paper applies the Dagum Gini coefficient approach. Along with the inter-regional relative differences and their evolutionary patterns, it also looks at the relative differences within China’s three main regions—Eastern, Central, and Western. The following formula is used to obtain the total Gini coefficient (G) [53–55]:


$$G=\frac{\sum_{j=1}^{k}\sum_{h=1}^{k}\sum_{i=1}^{n_{j}}\sum_{r=1}^{n_{h}}\left|y_{ji}-y_{hr}\right|}{2n^{2}\overset{―}{y}}$$ (12)

Where $y_{ji}$ and $y_{hr}$ denote the coupling coordination degree of digital economy and ecotourism of each province and city within the region, $\overset{―}{y}$ denotes the mean value of the coupling coordination degree, n denotes the number of provinces and cities, k denotes the number of divided regions, and $n_{j}$, $n_{h}$ denote the number of provinces and cities within the jth and hth regions.

The Gini coefficient $G_{jj}$ for area j is expressed as:


$${G_{j}}_{j}=\frac{\sum_{i=1}^{n_{j}}\sum_{r=1}^{n_{j}}\left|y_{ji}\text{-}{\text{y}}_{jr}\right|}{2n_{j}^{2}\overset{―}{y_{j}}}$$ (13)

The Gini coefficient $G_{jj}$ between areas j-h is expressed as:


$$G_{jh}=\frac{\sum_{i=1}^{n_{j}}\sum_{r=1}^{n_{h}}\left|y_{ji}\text{-}{\text{y}}_{hr}\right|}{n_{j}n_{h}(\overset{―}{y_{j}}+\overset{―}{y_{h}})}$$ (14)

(2) Kernel density estimation method

One non-parametric approach that effectively illustrates the evolving interaction between ecotourism and the digital economy is the kernel density estimation method.

This study makes the assumption that f(x) represents the density function of the coupling coordination degree X of the research variables [56–58]. The following is the probability density formula for the coupling coordination degree of ecotourism and the digital economy at a given place x:


$$f(x)=\frac{1}{Nh}\sum_{i=1}^{N}K\left(\frac{X_{i}-x}{h}\right)$$ (15)

Where N denotes the number of sample size, X_i_ denotes the level of coupled coordination of digital economy and ecotourism, x denotes the mean value of coupled coordination, K denotes the kernel density function, and h denotes the bandwidth.

(3) Standard deviation elliptic analysis

The concept of the standard deviation ellipse was introduced by Lefever [59]. It is employed to illustrate the distribution properties of geographic features in a two-dimensional space. The main and minor axes, as well as the center, are the primary quantitative characteristics that describe this ellipse. The following is the calculation formula [60,61]:

Centre of gravity of spatial distribution:


$$\overset{―}{X_{w}}=\frac{\sum_{i=1}^{n}w_{i}x_{i}}{\sum_{i=1}^{n}w_{i}},\;\overset{―}{Y_{w}}=\frac{\sum_{i=1}^{n}w_{i}y_{i}}{\sum_{i=1}^{n}w_{i}}$$ (16)

$$\sigma_{x}=\sqrt{\frac{\sum_{i=1}^{n}(w_{i}\overset{―}{x_{i}}\cos\theta -w_{i}\overset{―}{y_{i}}\sin\theta {)}^{2}}{\sum_{i=1}^{n}{w_{i}}^{2}}}$$ (17)

$$\sigma_{y}=\sqrt{\frac{\sum_{i=1}^{n}(w_{i}\overset{―}{x_{i}}\sin\theta -w_{i}\overset{―}{y_{i}}\cos\theta {)}^{2}}{\sum_{i=1}^{n}{w_{i}}^{2}}}$$ (18)

Where, $\overset{―}{X_{w}}$ and $\overset{―}{Y_{w}}$ represent the weighted centroid coordinates of the spatial distribution in the x and y directions, respectively; $\overset{―}{x_{i}}$, $\overset{―}{y_{i}}$ denote the relative coordinates of the centre of gravity of the spatial location $\left(\overset{―}{x_{i}},\overset{―}{y_{i}}\right)$ from the centre of gravity of the distribution, respectively; $W_{i}$ denotes the weight; $\sigma_{x}$ denotes the standard deviation along the x-axis and $\sigma_{y}$ denotes the standard deviation along the y-axis, θ is an angle parameter.

## Results

### Analysis of the Development Levels of the Digital Economy and Ecotourism

**Fig 1:**
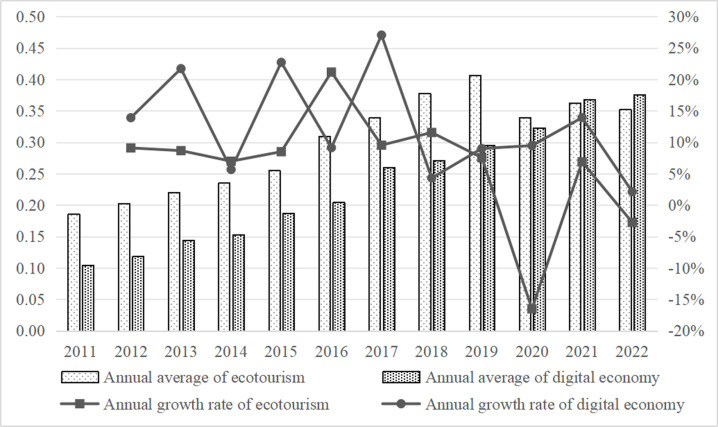
Annual averages and annual growth rates of the comprehensive development index for the digital economy and ecotourism.

Using the variables and weights given in Table 1, the annual average values and annual growth values for the comprehensive development indices of the digital economy and ecotourism were calculated for 30 provinces. The data shown in Fig 1 indicates that there were variations in the growth rates of the ecotourism development index and the digital economy development index between 2011 and 2022, but generally there was a continuous yearly increase. The COVID-19 pandemic in 2020 caused a decline in the ecotourism index.

**Fig 2:**
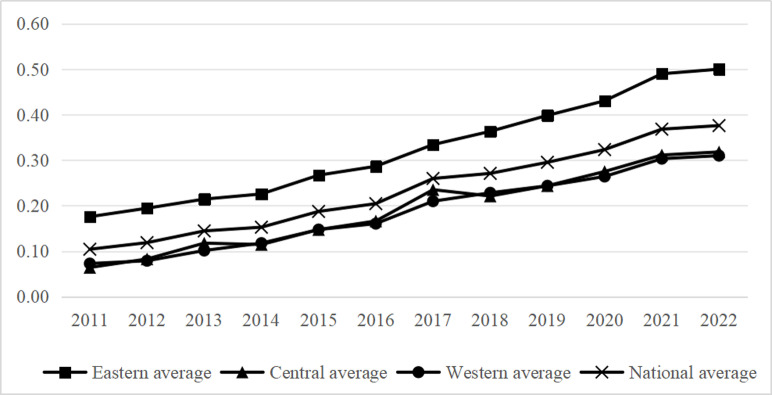
2011-2022 Trends in the level of development of the digital economy in three regions.

Figs 1 and 2 show that the size of China’s digital economy has grown by 3.6 times in terms of development pace. The digital economy development index for China as a whole, as well as for the eastern, central, and western regions, has continually maintained a stable upward trend, with the development momentum steadily rising, when looking at regional changes from 2011 to 2022. Notably, the western region experienced the largest increase in its digital economy.

**Fig 3:**
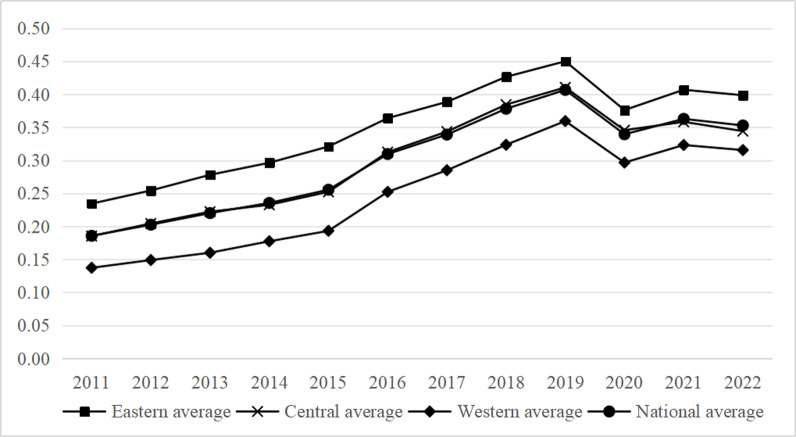
2011-2022 Trends in the level of ecotourism development in the three regions.

Fig 3 shows that, in terms of development speed, the average comprehensive indicator for the expansion of ecotourism in China has nearly doubled. China’s ecotourism, encompassing the eastern, central, and western areas, saw a period of growth followed by a fall between 2012 and 2022. Nevertheless, the general trajectory of development remained favorable, with the eastern region witnessing the most substantial growth in the development of ecotourism.

### Analysis of coupling coordination results

**Fig 4:**
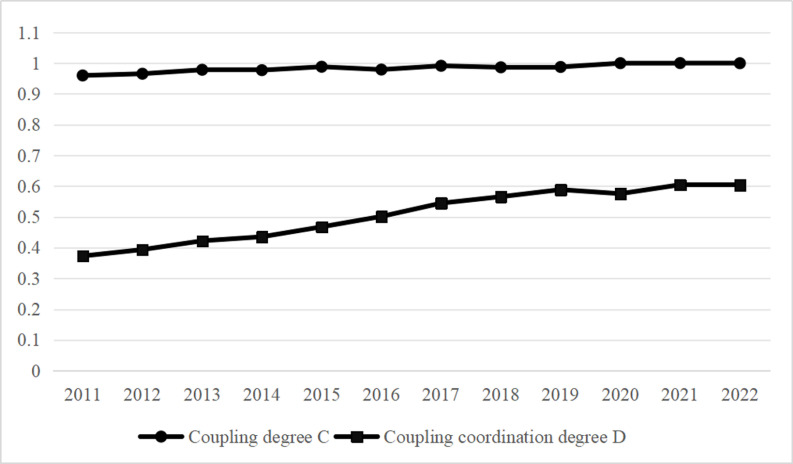
Trend chart of coupling degree and coupling coordination degree changes for digital economy and ecotourism from 2011 to 2022.

China’s ecotourism and digital economy have developed in tandem, as shown in Fig 4, however there is still a noticeable gap in comparison to ideal coordination.

**Fig 5:**
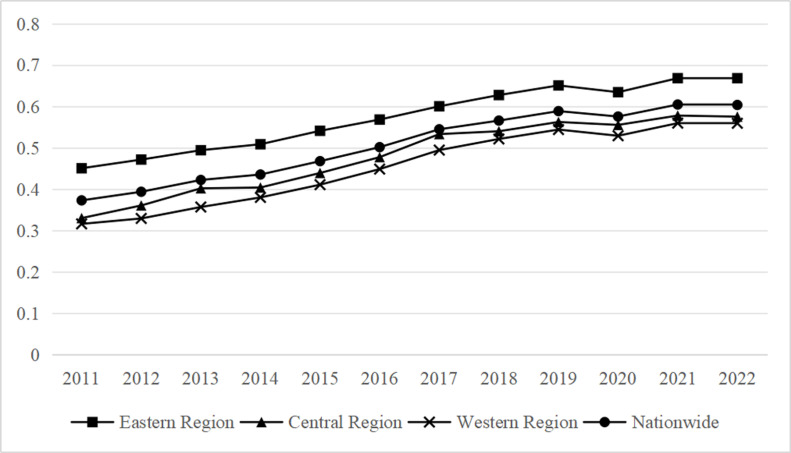
The coupling coordination degree of digital economy and ecotourism in the eastern, central, and western regions.

As can be shown in Fig 5, there was a positive growing trend in 2022 in the coupling coordination degree between China’s ecotourism systems and digital economy. However, the COVID-19 pandemic’s impact caused it to decline in 2020. The three primary zones show considerable differences in coupling coordination, with the eastern region showing the highest amount of coupling coordination. There is still a noticeable difference in the amount of coordination when compared to the eastern area, even if the central and western regions exhibit more positive development tendencies.

**Fig 6:**
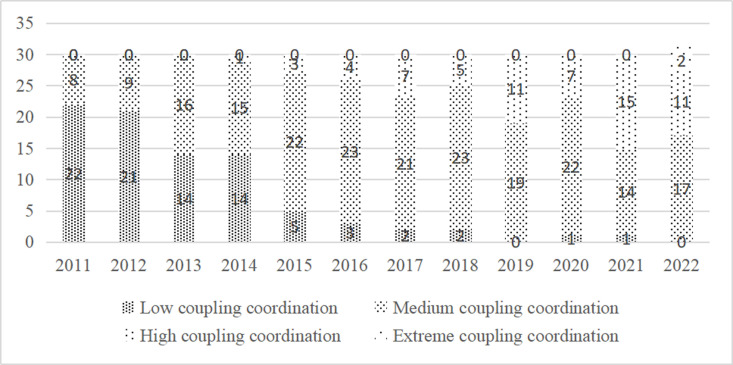
The temporal evolution trend of coupling coordination types between digital economy and ecotourism in China’s provincial regions.

The degree of connection coordination between the digital economy and ecotourism is carefully classified using the classification criteria listed in Table 2. The growth of coupling coordination types over time at various places is seen in Fig 6. The data illustrated in Fig 6 indicates a continuous and steady increase in the level of cooperation between China’s ecotourism and digital economy between 2011 and 2022. The degree of linking development has changed in the majority of provinces in a “transitional” way. The level of cooperation between China’s digital economy and ecotourism has increased from a low to a medium stage in 2019.

### Examining the spatial and temporal changes in the degree of linkage coordination

#### Decomposition of regional differences in the degree of coupling coordination.

This study properly analyzes China’s regional disparities and their causes by using the Dagum Gini coefficient and its decomposition approach. It computes the contribution rates of various areas in various years, as well as the overall Gini coefficient and intra- and interregional disparities. The particular outcomes are listed in Table 3.

**Table 3:** Results of the decomposition of the overall Gini coefficient and the Gini coefficient for the three regions.

Year	Overall Gini Coefficient	Intra-regional Gini Coefficient			Inter-regional Gini Coefficient			Contribution %		
		East	Central	West	East- Central	East- West	Central-West	Intra- region	Inter- region	Super- variable Density
2011	0.131	0.098	0.056	0.105	0.156	0.185	0.089	24.19	64.11	11.70
2012	0.120	0.090	0.038	0.086	0.129	0.184	0.087	22.32	70.85	6.83
2013	0.107	0.090	0.030	0.072	0.108	0.167	0.080	22.83	70.78	6.39
2014	0.102	0.097	0.028	0.071	0.118	0.151	0.059	25.03	65.21	9.77
2015	0.101	0.098	0.033	0.076	0.111	0.145	0.065	26.26	62.36	11.38
2016	0.096	0.087	0.033	0.090	0.097	0.135	0.074	27.31	58.30	14.39
2017	0.091	0.084	0.035	0.086	0.084	0.125	0.078	27.92	52.73	19.35
2018	0.094	0.089	0.038	0.088	0.095	0.128	0.071	28.35	54.15	17.49
2019	0.092	0.089	0.050	0.086	0.093	0.117	0.074	29.77	46.58	23.65
2020	0.088	0.089	0.044	0.079	0.089	0.114	0.069	29.57	48.64	21.79
2021	0.088	0.087	0.052	0.079	0.092	0.112	0.070	29.66	47.57	22.76
2022	0.095	0.099	0.053	0.084	0.100	0.119	0.074	30.24	44.09	25.67

(1) Table 3 shows the overall relative differences and their changes over time. From a national standpoint, the gaps in the coordinated development levels of China’s digital economy and ecotourism fluctuated downward between 2011 and 2022. The Gini coefficient saw a fall from 0.131 in 2011 to 0.091 in 2017, with a particularly significant decrease observed between 2011 and 2014. Despite seeing a rise in 2018, the value declined to 0.088 by 2021, only to rebound in 2022. Overall, there has been a steady decline in regional disparities in the coordinated growth of ecotourism and the internet economy. The use of regional coordinated development, the “Eastern Data for Western Computing” project, and the growth of China’s western regions are examples of coordinated development initiatives that have greatly enhanced The uneven development of the digital economy and ecotourism in various areas is out of balance.
(2) Analysis of Intra-regional Relative Differences and Their Evolution. The eastern region’s Gini coefficient showed a trend of declining, increasing, and then decreasing again over the research period, making it noticeably bigger than that of the central and western areas. When it comes to promoting synergy between the digital economy and ecological tourism, the central and western areas have the greatest degree of discrepancy, while the center region has relatively less variances. Crucially, over the course of the study period, the Gini coefficients for inter-regional disparities in the three major regions showed a steady upward trend, indicating the existence of imbalanced development in the concurrent expansion of the digital economy and ecological tourism across different regions. This underscores the ongoing need to address issues related to coordinated development at the regional level.
(3) Factors causing variations and rates of contribution. Various sources of disparities exhibit divergent patterns. The rate of contribution from intra-regional differences has exhibited a consistent upward trend, growing from 24.19% in 2011 to 30.24% in 2022. The contribution rate of the super-variable density exhibits an annual growth, seeing a substantial surge from 11.70% in 2011 to 25.67% in 2022. Conversely, the proportion of inter-regional disparities in contributions has experienced a significant decline, dropping from 64.11% in 2011 to 44.09% in 2022. Although there has been a significant decrease in the extent to which inter-regional differences contribute to the regional development imbalance in the digital economy and ecological tourism, it still remains the primary component, while intra-regional differences play a minor role. An increasing contribution rate for intra-regional and highly variable density variations is revealed by the examination of the three causes of differences. On the other hand, the inter-regional disparities’ yearly contribution rate is declining. This implies that regional coordinated development initiatives are beginning to show some signs of success, and that the gap between ecological tourism and regional digital economies in terms of coordinated development is closing. However, the issue of unequal growth among areas still makes it necessary to continue implementing and growing strategic plans meant to encourage collaboration.
(4) Origins and Magnitudes of Variability. Various sources of variability display unique patterns. The intra-regional variability exhibits a consistent upward trend, rising from 24.19% in 2011 to 30.24% in 2022. The contribution rate of hyper-variable density exhibits an annual rising trend, with a notable surge from 11.70% in 2011 to 25.67% in 2022. In the meantime, inter-regional variability’s percentage has dropped significantly, from 64.11% in 2011 to 44.09% in 2022. Over the course of the research period, inter-regional variability has remained the major component, despite a decline in its contribution rate. Hence, the primary causes of the regional growth imbalance in the digital economy and ecological tourism are the variations between different regions, while the disparities within a region play a minor role. Upon analyzing the patterns of these three sources of variability, it is evident that the rates at which intra-regional and hyper-variable density differences contribute are increasing, while the rate at which inter-regional differences contribute is decreasing each year. These findings indicate that the disparity in the progress of coordination development between regions in the digital economy and ecological tourism is gradually narrowing, suggesting that the strategy for regional coordinated growth has been fairly successful. However, the problem of uneven growth between areas still has to be addressed and requires the adoption and enhancement of policies for coordinated development at the regional level.

#### Analysis of the time-series evolution of the coupling coordination degree.

**Fig 7:**
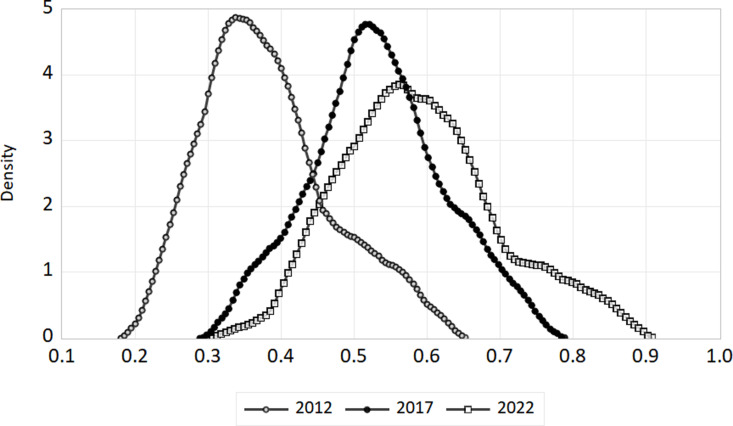
Kernel density in nationwide.

**Fig 8:**
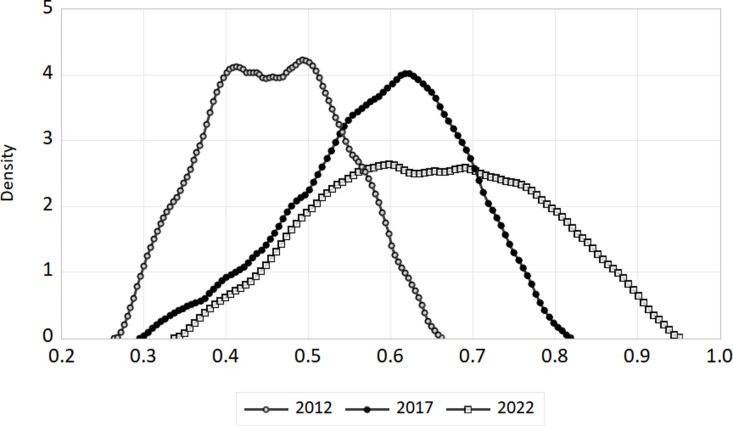
Kernel density in eastern region.

**Fig 9:**
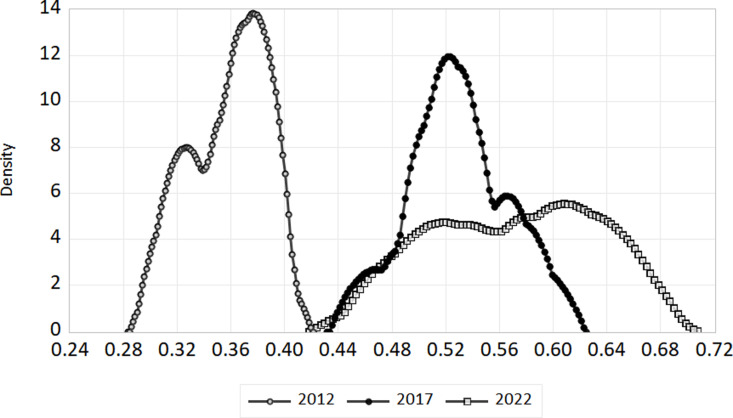
Kernel density in central region.

**Fig 10:**
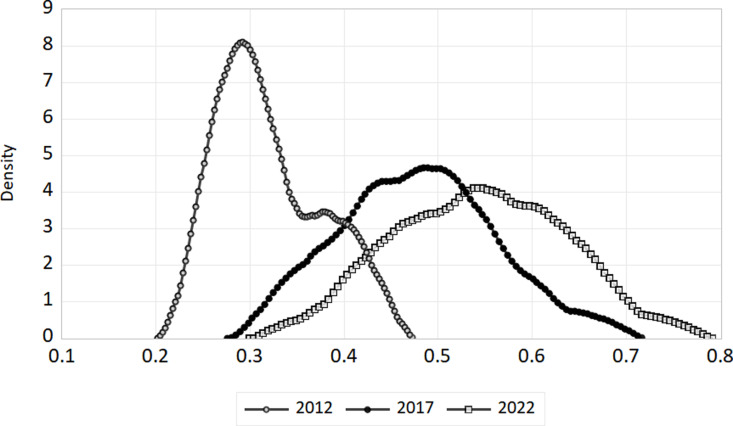
Kernel density in western region.

We’ve selected 2012, 2017, and 2022 as benchmarks to illustrate how the digital economy and ecotourism are evolving together. For the whole nation as well as the eastern, central, and western areas, we have plotted kernel density curves. The Figs 7–10 show these trends.

(1) National level. The link between China’s digital economy and the spread of ecological tourism evolves between 2012 and 2022, as shown in Fig 7. The coupling coordination kernel density curve shifts to the right, indicating a steady improvement in the coupling coordination between ecological tourism and the digital economy at the national level. From 2012 to 2017, the distribution’s morphology remained mostly unchanged, with a constant peak height and no significant changes to the peak breadth. This suggests that coupling coordination dispersion is essentially constant. The right tail’s yearly elongation signifies a steadily growing geographical disparity between the nation’s coordination of the digital economy and ecological tourism.
(2) Geographical Level. The distribution of coupling coordination degrees in the eastern, central, and western regions of China changed between 2012 and 2022, as shown in Figs 8–10. For each of the three sites, the kernel density distribution curves indicate a progressive movement from left to right, indicating a steady augmentation. About how closely connected and coordinated the internet economy and ecotourism are in different places. In both the eastern and central areas, there was a discernible rise in the breadth of peaks and a constant drop in the maximum height of peaks over the research period. This points to a definite increase in coupling coordination dispersion. Conversely, the peak’s width initially widens and then compresses, while the greatest height in the center area first decreases and then increases. This suggests that there is some variance in the degree of coupling coordination dispersion in this area. The kernel density distributions of the eastern and western sections display a unimodal distribution, whereas the central region transitions from a unimodal to a bimodal distribution, indicating a distinct polarization tendency. There is no evidence of a substantial tailing phenomenon in any of the three zones, suggesting that the distribution is steady.

#### Analysis of the spatial evolution of coupling coordination.

We used the years 2012, 2017, and 2022 as reference points to look at how the spatial distribution of the degree of cooperation between the digital economy and ecotourism has changed over time. Next, we drew standard deviation ellipses for the whole nation as well as the eastern, central, and western areas. The key parameters of these standard deviation ellipses for each year are provided in Table 4.

**Table 4:** Standard deviation ellipse parameter of coupling coordination degree of digital economy and ecotourism in China.

Region	Year	Area/km^2^	X-axis coordinates	Y-axis coordinates	Long half-axis/km	Short half-axis/km	Azimuth (°)
Nation- wide	2012	3467416.383	113°27′	33°79′	1148.219	935.605	26.786
	2017	3457485.707	112°94′	33°74′	1147.127	946.126	29.453
	2022	3505632.644	112°76′	33°56′	1145.906	963.684	29.139
Eastern	2012	1329052.392	117°72′	32°80′	1114.025	379.818	7.280
	2017	1296667.823	117°73′	32°92′	1099.508	375.455	6.508
	2022	1319146.194	117°65′	32°70′	1114.521	376.820	7.341
Central Region	2012	1324607.888	117°02′	35°35′	1279.765	329.562	22.862
	2017	1345500.101	116°89′	35°18′	1261.934	339.483	22.478
	2022	1316732.862	116°83′	34°96′	1241.354	337.730	22.271
Western	2012	2655665.447	103°89′	33°65′	1068.084	791.486	153.502
	2017	2607564.204	103°93′	33°31′	1071.790	774.464	155.143
	2022	2661275.191	103°84′	33°22′	1086.758	779.530	155.015

(1) Key features may be seen in the regional dynamics of the coupling coordination degree between ecological tourism and the digital economy from 2012 to 2022. Generally speaking, the standard deviation ellipse points from the northeast to the southwest. Over the course of the research period, the centroid of coupling coordination moved from a place with coordinates of 113°27’E, 33°79’N to a new site with values of 112°76’E, 33°56’N. The elliptical area’s fluctuating expansion suggests a dispersion pattern in the linking coordination between China’s ecotourism and digital economy. The standard deviation ellipse’s angle of orientation increased between 2012 and 2017, then somewhat decreased by 2022. This suggests that there was a general trend of increasing and then stabilizing spatial distribution orientation in the coupling coordination degree.
(2) Two Considering the viewpoint of a specific geographical area: Upon analyzing Table 4, it is evident that the centroid of coupling coordination in the eastern region throughout the study period was situated inside Anhui Province, specifically within the coordinates 117°65’E and 117°73’E longitude, and 32°70’N and 32°92’N latitude. By observing the trajectory of the centroid movement, it can be seen that it first shifted towards the north and then moved towards the southwest, with the north-south movement being greater than the east-west movement. The standard deviation ellipse’s orientation revealed an increasing tendency over the research period in both the north and south, indicating a coupling coordination between the digital economy and ecological tourism in the eastern area of China. Indicating a tendency towards higher geographic dispersion in coupling coordination in the eastern region, the standard deviation ellipse first shrank before growing. A constant route of alignment between the digital economy and ecological tourism in the eastern area is shown by the modest fluctuation in the orientation angle of the standard deviation ellipse.

It is evident from the data in Table 4 that over the research period, the centroid of coupling coordination in the center region moved from coordinates of 117°02’E, 35°35’N to 116°83’E, 34°96’N. The majority of the centroid is located in Shandong Province. The standard deviation ellipse displayed a geographical distribution that typically stretched from northeast to southwest, although the centroid shifted in a southwesterly manner. Indicating an improvement in the geographical clustering of coupling coordination between the digital economy and ecological tourism in the center region, the standard deviation ellipse first extended and then shrunk.

Based on Table 4, the centroid of coupling coordination in the western region was located inside Ningxia Province, specifically between the longitudes 103°84’E and 103°93’E, and the latitudes 33°22’N and 33°65’N, during the study period. The centroid initially shifted southward and subsequently moved towards the southwest, with the southerly migration becoming increasingly noticeable. The ellipse representing the standard deviation was positioned from northwest to southeast. The standard deviation ellipse’s size decreased and subsequently increased, while its orientation angle increased, rising from 153.502° in 2012 to 155.015° in 2022. The development gap between the eastern and western areas must be addressed, and in the future, provinces with strong coupling coordination along the east-west axis must be given more prominence and influence.

## Discussion

The digital economy is crucial for the sustainable development of ecotourism, and achieving a higher degree of coupling coordination can contribute to the high-quality development of both the economy and culture. Firstly, previous studies have focused on the heterogeneous effects of spatial and temporal evolution, overlooking the spatial-temporal evolutionary trends of coupling coordination across different regions [62,63]. Secondly, existing literature tends to adopt a singular perspective, focusing solely on the static analysis of coupling coordination [64–66]. In contrast, this paper reveals the dynamic spatial-temporal evolution patterns between the digital economy and ecotourism, enriching the existing research perspectives and providing a reference for accurately quantifying their coordination level to assess spatial-temporal evolution trends.

However, due to the limitation of information and data, the research in this paper can not be exhaustive and will continue to be discussed in depth in the future. (1) Considering the availability of sample data, there is subjectivity in the selection of indicators for digital economy and ecotourism, which may result in the constructed indicator evaluation system not being comprehensive enough, therefore, this paper lacks in the construction of the indicator system, and the indicator system of digital economy and ecotourism will be further improved in the future. (2) Existing literature on the coupled and coordinated development of digital economy and ecotourism has less research, and fewer references may lead to the influence factors considered are not comprehensive enough, which will cause some bias to the conclusions drawn, and other key influence factors will be further explored in the future.

The study finds that there is a low degree of coordination and significant regional disparities between the development of the digital economy and ecotourism in China. There are two main regional differences in the coupling coordination of the digital economy and ecotourism: First, inter-regional disparities, where the eastern regions clearly outperform the western regions. The eastern regions should play a radiating and guiding role for the west, while the western regions must strengthen their initiative and proactivity. Second, intra-regional disparities, where the central region has the lowest Gini coefficient and the best coordination status. The central region should uphold a “unified strategy” approach and play a “bridge” role. On a broader scale, some provinces exhibit significantly higher coupling coordination than others, with a clustering pattern in their spatial distribution. These provinces should integrate into regional development strategies and explore the potential for the development of both the digital economy and ecotourism.

1. The eastern region has a substantial digital industry and a high-quality ecological tourism development level, thus it should actively exert its radiating and driving effects, facilitating the digital transformation of ecological tourism from the east to the central and western regions while providing robust support to others. This involves promoting the penetration of digital technologies and advancing the “Internet + Ecology” paradigm. The modernization and digitization of ecological conservation and management are facilitated through the support of digital technologies. For instance, internet platforms are used to collect and analyze ecological data, while intelligent technologies promote the efficient use and protection of ecological resources. Specifically, it should accelerate the digital transformation of traditional tourism services, support businesses in creating quality digital content and platforms, and establish information-sharing platforms to optimize collaboration with the western region. Additionally, it should encourage the penetration of various new business formats and models into ecological tourism, utilizing VR/AR, AI, and drones to develop new “Internet + Exhibition” models and enhance the digital display industry.
2. The western region, positioned relatively peripherally, lacks a solid digital industry and tourism foundation, limiting its ability to attract capital and talent, and consequently its impact on other regions. Thus, it must proactively deepen cooperation with the eastern region, seizing opportunities in the digital era to establish comprehensive, multi-level cooperative mechanisms. This includes enhancing policy support for digital tourism innovations, accelerating the construction of digital infrastructure, and maximizing local ecological tourism resources to convert ecological advantages into economic benefits, paving the way for green, low-carbon ecological tourism development.
3. The intra-regional disparities highlighted by the Gini coefficient necessitate urgent resolution. Occupying a central position in the spatial structure of the digital economy and ecological tourism, the central region should actively play a regulatory role between the eastern and western regions, fostering cooperation, optimizing resource allocation, and reducing administrative obstacles to establish a new digitalized ecological tourism landscape. Establishing interdependent partnerships through collaboration among local stakeholders, policymakers, and NGOs will help to implement sustainable development concepts and create inclusive, mutually beneficial digital ecological tourism strategies.
4. From a broader perspective, the spatial disparities between China’s digital economy and ecological tourism are gradually diminishing, with the three major regions exhibiting distinct clustering trends and development trajectories. Therefore, integrating into regional development strategies is essential. This includes building digital tourism industrial clusters and encouraging the concentration of digital tourism industries in key tourism development platforms such as national ecological tourism demonstration zones and cultural industry innovation pilot areas, aligning the development of digital tourism with national strategic emerging industries and demonstrating the integration of digital and tourism sectors.
5. The increasing dependency of China’s three major regions on the digital economy and ecological tourism indicates that the relationship between them has not yet reached its ideal state, presenting substantial room for growth. First, policies can utilize digital technologies to enhance the flexibility of natural resource conservation policies and provide opportunities for addressing ecological tourism land-use restrictions. Second, big data can be employed to broaden promotional pathways for various ecological tourism models. Third, aligning with the trends of ecological compensation and payment, a government-led, data-driven approach can effectively achieve the rational allocation of ecological resources and the value enhancement of ecological products. Lastly, as digital economy trends emphasize data privacy, security, and ethics, it is crucial to promote responsible and ethical digital transformations in ecological tourism. Accelerating the integration of the two sectors is of significant contemporary relevance for achieving the digital transformation of ecological tourism and promoting the healthy development of the tourism industry.

## Conclusion

The purpose of this article is to present an indicator system for evaluating the digital economy and ecotourism in mainland China’s thirty provinces and municipalities—excluding Tibet. The degree of coordination and connection between two entities is measured using the coupling coordination degree model. The technique investigates the spatiotemporal evolution elements of the coupling coordination degree using the Dagum Gini coefficient, kernel density estimation, and standard deviation ellipse analysis. The results of the investigation indicate: (1) Both the digital economy and ecotourism have consistently advanced in terms of development between 2011 and 2022. Regional disparities exist in the growth of these industries, nevertheless, with the eastern region outpacing the western region in the development of the digital economy and ecotourism. (2) According to the coupling coordination degree model, the coupling degree between ecotourism and the digital economy remained above 0.9 from 2011 to 2022, indicating strong interdependence. The coordination degree increased from below 0.4 to 0.6, gradually reaching a high coordination state. Regional disparities persist, with the eastern region demonstrating better coordination than the western region, mainly due to inter-regional differences. (3) The overall inequalities of the coupling coordination degree appear to be decreasing, according to the results of the Gini coefficient. The center region’s intraregional Gini coefficient is significantly lower than that of the eastern and western regions, highlighting the urgent need to address the issue of unequal development within regions. The disparities between various regions are the main source of variance. (4) The kernel density estimate shows a steady national increase in coupling coordination. The east and west remain “unimodal,” while the central region shifts to “bimodal,” indicating polarization. The national curve’s right tail rises annually. (5) The standard deviation ellipse analysis reveals that China’s digital economy and ecotourism align northeast-southwest. The east trends north-south, the center northeast-southwest, and the west northwest-southeast. Spatial inequalities decrease, evidenced by shorter ellipses and increased clustering.

## Supporting information

S1 Fig Annual averages and annual growth rates of the comprehensive development index for the digital economy and ecotourism. (XLSX)

S2 Fig The level of development of the digital economy in three regions. (XLSX)

S3 Fig The level of ecotourism development in the three regions. (XLSX)

S4 Fig Coupling degree and coupling coordination degree changes for digital economy and ecotourism from 2011 to 2022. (XLSX)

S5 Fig The coupling coordination degree of digital economy and ecotourism in the eastern, central, and western regions. (XLSX)

S6 Fig The temporal evolution trend of coupling coordination types between digital economy and ecotourism in China’s provincial regions. (XLSX)

S7 Fig Original data on Kernel density in nationwide. (XLSX)

S8 Fig Original data on Kernel density in eastern region. (XLSX)

S9 Fig Original data on Kernel density in central region. (XLSX)

S10 Fig Original data on Kernel density in western region. (XLSX)

S1 Table Original data. (XLSX)

S2 Table Entropy weight method for raw data weighting. (XLSX)

S1 File Composite index of digital economy development (selected years). (XLSX)

S2 File Composite index of ecotourism development (selected years). (XLSX)
